# Primary pulmonary mucinous cystadenocarcinoma presenting as a complex bronchocele: a case report

**DOI:** 10.4076/1752-1947-3-8581

**Published:** 2009-08-07

**Authors:** Syed Arsalan Raza, Christopher Alexakis, Michael Creagh, David R Lawrence, Michael Wood

**Affiliations:** 1 Department of Radiology, St George's Hospital NHS Trust, London, SW17 7NS, UK; 2 Department of Radiology, Ashford & St Peter's Hospital NHS Trust, Guildford Road, Chertsey, KT16 0PZ, UK; 3 Department of Respiratory Medicine, Ashford & St Peter's Hospital NHS Trust, Guildford Road, Chertsey, KT16 0PZ, UK; 4 Department of Cardiothoracic Surgery, University College London Hospital, Euston Road, London, NW1 2BU, UK

## Abstract

**Introduction:**

Primary pulmonary mucinous cystadenocarcinoma is a rare variety of lung cancer. It is characterized pathologically by copious mucin production predominantly in the extracellular space. This tumour has a remarkably favorable prognosis.

**Case presentation:**

We present imaging and histopathological findings of primary pulmonary mucinous cystadenocarcinoma presenting as a complex bronchocele in a 67-year-old Caucasian woman.

**Conclusion:**

Diagnosis of pulmonary mucinous cystadenocarcinoma should be considered in patients presenting with bronchocele that has suspicious imaging features, because the results of fine needle aspiration cytology and bronchoscopy are frequently inconclusive in these tumours. Positive emission tomography has an important role in helping to identify these tumours.

## Introduction

Primary pulmonary mucinous cystadenocarcinoma (PMC) is a rare variety of lung cancer. It is characterized pathologically by copious mucin production predominantly in the extracellular space. This tumour has a remarkably favorable prognosis.

We present imaging, which includes positive emission tomography (PET-CT), and histopathological findings of a primary PMC presenting as a complex bronchocele. Diagnosis of pulmonary mucinous cystadenocarcinoma should be considered in patients presenting with a bronchocele that has suspicious imaging features, since the results of fine needle aspiration cytology and bronchoscopy are frequently inconclusive.

## Case presentation

A 67-year-old Caucasian woman presented to our emergency department after she had collapsed. She was found to be hyponatraemic. Her medical history consisted of hypertension, previous fractured left neck of femur and bilateral breast implants for cosmetic reasons. She had a 50 pack year smoking history but had given up six months previously. She described a more insidious history of tiredness and weight loss of 2 kg over the preceding few months, although she denied having coughs or haemoptysis. Physical examination of all systems including her chest was unremarkable.

**Figure 1:**
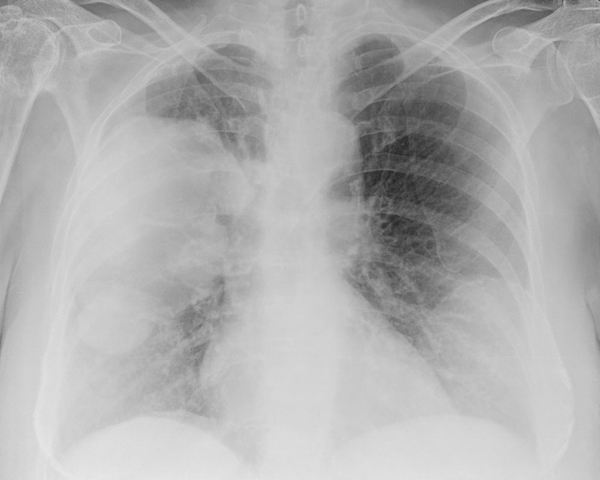
**Chest X-ray showing a large opacity in the right hemithorax**.

**Figure 2:**
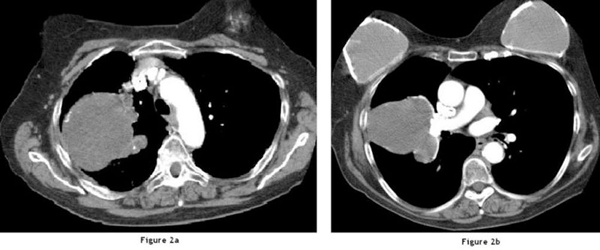
**Computed tomography of the thorax at the level of aortic arch**. (**Left**) This image shows a large cystic lesion in the right hemithorax with communication with the subsegmental dilated bronchi ('fingers in gloves' appearance). (**Right**) This image shows a section more caudally demonstrating enhancement within the medial wall of the lesion. Note is made of bilateral breast implants.

**Figure 3:**
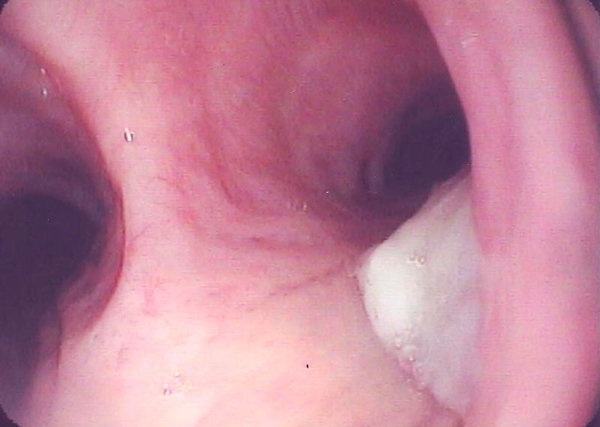
**Bronchoscopic view of the carina with the mass protruding from the right upper lobe bronchus**.

Her chest X-ray showed a large soft tissue density lesion in the right hemithorax, which included her bilateral breast implants (Figure 1). A computed tomography (CT) of her chest showed a large mass, predominantly in the right upper lobe of the lung extending from the right hilum. The mass demonstrated a largely cystic appearance (Hounsfield unit 14) communicating with the sub-segmental dilated bronchi that gave a 'fingers in glove' appearance (Figure 2A). There was a notable mural enhancement of the medial aspect of this mass adjacent to the right hilum (Figure 2B). A bronchoscopy revealed a soft tissue lesion in the right upper lobe bronchus with the appearance of granulation or necrotic tissue (Figure 3). At that time, a provisional diagnosis of complex bronchocele based on CT findings was made.

**Figure 4:**
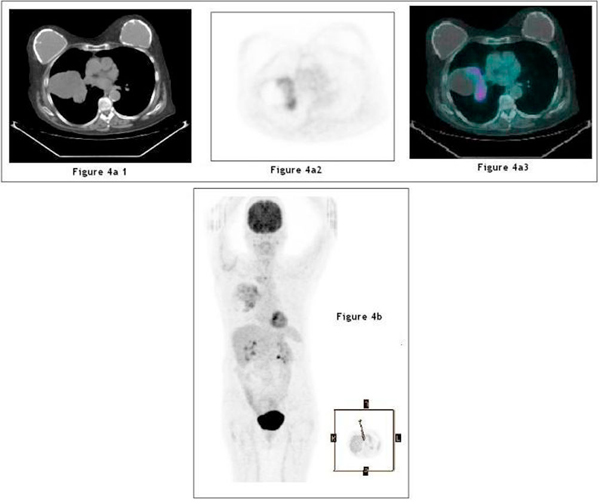
**(**a**) Positive emission tomography images showing avid fludeoxyglucose uptake within the medial aspect of the cystic lesion**. (**b**) Maximum intensity projection image of the positive emission tomography demonstrating the extent of the abnormal uptake within the lesion.

**Figure 5:**
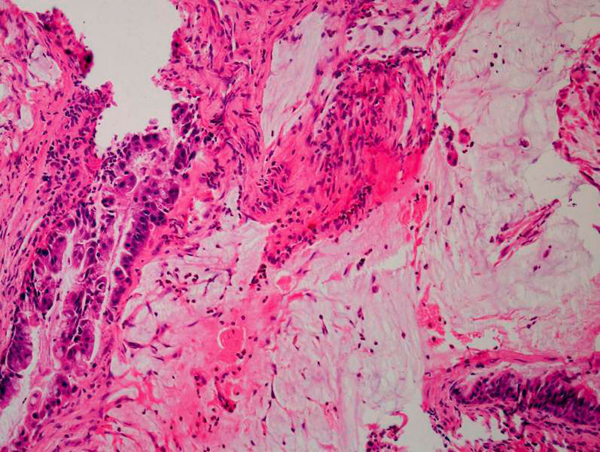
**Histopathology slide showing pools of mucin within the extracellular space with malignant cells predominantly on the left side**. Scattered malignant cells are seen within the mucin pool.

A PET-CT scan was subsequently performed, which demonstrated fludeoxyglucose uptake within the medial side of the mass with an SUV of 4 to 5 (Figure 4A). No other abnormally active areas were identified in the chest or elsewhere (Figure 4B). The findings strongly suggested malignant pathology, so the patient was referred to cardiothoracic surgeons for an open lung biopsy. Operative findings revealed a complex cystic mass involving all three lobes of the right lung. Histopathology showed pools of mucin within the extracellular fibroconnective tissues with scattered pleomorphic malignant cells within the mucin pool. Immunocytochemistry confirmed the diagnosis of a primary lung mucinous cystadenocarcinoma (Figure 5).

Curative surgery was not attempted in view of extensive multilobar involvement of the tumour and poor pre-operative lung reserve. The patient was referred to oncologists for radical radiotherapy with the possibility of chemotherapy.

## Discussion

Pulmonary mucinous cystadenocarcinoma (PMC) now represents a distinct but rare clinico-pathological entity. It was first described in 1978 by Gowar [1] and since then only 20 cases have been reported [2]-[5]. They are considered to be the pulmonary counterpart of similar tumours in the stomach, colon, ovaries and pancreas.

PMCs are described as well-demarcated peripheral cystic tumours and are microscopically characterized by copious mucus production that is mainly extracellular and exhibiting only a small number of cancer cells in the periphery of the lesion.

CT characteristics of PMC described in the literature include a uniform low attenuation cystic lesion with focal thickening and enhancement of the walls and septa [6]. The radiological differential diagnosis of a cystic lesion in the lung includes congenital causes (pulmonary sequestration, bronchogenic cyst or bronchocele due to bronchial atresia), infection (hydatid cyst or abscess) and neoplasm (pulmonary metastases from mucinous adenocarcinoma). A thick-walled cystic lesion particularly with focal mural or septal enhancement should raise the suspicion of PMC. The paucity of cancer cells within the lesion means that fine needle aspiration and bronchoscopy are frequently non-diagnostic [5] and a strong degree of suspicion is usually based on radiological findings.

PMCs, as with most lung cancers, predominantly occur in older age (age range 41 to 71 years; average of 59 years), with a positive smoking history and presentation similar to bronchogenic carcinoma. However, the biologic behaviour of these tumours is remarkably favorable with excellent prognosis described after curative surgery [6], hence the need for accurate diagnosis.

The pathogenesis of the bronchocele in this patient could have been due to a neoplastic lesion causing focal obstruction of the central bronchi leading to failure of the mucus drainage. Moreover, the tumour itself may cause an absolute increase in mucus production leading to stretching and destruction of airspaces and consequent cyst formation [5]. This also explains the potential for rapid expansive growth in these tumours. This mode of radiological presentation has been described for other lung cancers. Aronberg et al. [7] described three cases of small-cell lung carcinoma presenting as bronchocele.

## Conclusion

In summary, we presented a rare but recognized case of primary lung cancer. Diagnosis of primary PMC should be kept in mind when investigating a bronchocele with suspicious features, especially if bronchoscopy and fine aspiration cytology results are inconclusive. Moreover, this case illustrates the important role of PET-CT in helping to differentiate and identify areas that may be malignant within a tumour.

## Abbreviations

PET-CT: Positive Emission Tomography - Computed Tomography; PMC: Pulmonary Mucinous Cystadenocarcinoma; SUV: Standardized Uptake Value.

## Consent

Written informed consent was obtained from the patient for publication of this case report and any accompanying images. A copy of the written consent is available for review by the Editor-in-Chief of this journal.

## Competing interests

The authors declare that they have no competing interests.

## Authors' contributions

SAR and MC were involved in the radiological assessment of the patient. CA, DRL and MW were involved in clinical care. SAR wrote the radiological discussion contained in the manuscript. CA wrote the clinical presentation. MC, DRL and MW reviewed and edited the manuscript. All authors read and approved the final manuscript.
